# Supplementary material for: Chromothripsis during telomere crisis is independent of NHEJ, and consistent with a replicative origin
Source: Genome Res. 2019 May;29(5):737–49. doi: 10.1101/gr.240705.118 (PMC6499312; doi:10.1101/gr.240705.118)
Supplement: Supplemental Material [file supp_gr.240705.118_Supplemental_file_1.zip › contigs/annotated_contigs/DB49/contig.2.DB49_length_788_mean_cov_12.0596446701.docx]

**DB49_length_788_mean_cov_12.0596446701**

```
TCTTTATATCCTCTGATTAACAATTTTCATTAGTAATGTGTTACACAACCAGTACTTGTTTAGACTAACCTACATTTTACCAATTTCTT
 >chr10:38984295-38985018 - E=0e+00 p=2e-02
TGCACATTGTTCCTTCCTTTTTGGTTCTATGTCATATTTCTAAAGCATATCTTTCAGTAGATTTTTCCCTGGTAAAAAATGGCATATGT

TCTTAAATGTATTTGTTTGACAATATCTTTCATTTGTCTTTTGTTTGAGTGACAGTTAACTGGCTACAGAATTCAACATTGAATGATTT

CTTTATCTCTGTACTTTGAAGACTTTATTTATCTTCCATCTTCTATTGTTGCTGATAAGTACTGAGAATCTGCTCTATTGTTTATTCGA

TTATGATTAATCTCTTTTTCTCTGTCTAGATTCTTTAAGATTTTATTTTTGCCTTTGGTAGTCTATAGTTTCTCAACATTTTATCTAAG

TATCTATTTTTACTTAATCCTGATTAGAACTCATTTGACTTCCTGTGTCCAAATCAATAAAAACCCTAATCAATTCTGTAAAGTTTGAA

GCCAGTTTTCTTCTCAAATATTATATCTTCTTCATTCTTTCAATTATGTTTCTGAAACTACTATTAGTTGTGTTTTAGATCTTTTCCAT

AATCTAAATCTCTCATTTTCCACCTCATTTTTTTTTGTGTGTGTGCTATGCTCTTAAGTAATTTCCTCATATCTACTACTTTGCTGTGT

TCTCTCTTC|TGCCATTTCTCGACTGTTATTTGGTT|TTTTATTGGTTTTTTTTTTATTTTATTTTATTTT|TCTGTTT                                    >chr10:80254970-80255004 - E=1e-02
```
